# Nasal cavity changes and the respiratory standard after maxillary expansion

**DOI:** 10.1016/S1808-8694(15)31388-4

**Published:** 2015-10-17

**Authors:** Tatiana Ramires, Roberto Alcântara Maia, José Roberto Barone

**Affiliations:** 1 Specialist in Buccomaxillofacial Surgery and Traumatology. Master’s degree student in Health Sciences, Hospital do Servidor Público Estadual de São Paulo; 2 Doctor, otorhinolaryngologist, Hospital do Servidor Público Estadual de São Paulo; 3 Specialist in Buccomaxillofacial Surgery and Traumatology. Dental surgeron. Assistant of the Buccomaxillofacial Surgery and Traumatology Unit, Hospital do Servidor Público Estadual de São Paulo. Hospital do Servidor Público Estadual de São Paulo

**Keywords:** nasal cavity, cephalometry, maxilla, palatal expansion technique

## Abstract

Mandibular cross-sectional deficiency is a dentofacial defect in connection with the narrowing of the mandibular arch width. This abnormality is a significant etiopathogenic factor and it is often associated with nasal breathing difficulties. This atresia may be treated through Rapid Maxillary Expansion or Surgically Assisted Rapid Maxillary Expansion, depending on the patient’s age. Both procedures will change the craniofacial structure, especially the nasal cavity.

**Aim:**

Based on literature review, the purpose of this paper was to report the relationship among maxillary expansion, nasal cavity and Nasal Airflow Resistance.

**Method:**

A non-systematic literary review was conducted in search of experimental studies to treat maxillary atresia. Papers considering Rapid Maxillary Expansion and Surgically Assisted Rapid Maxillary Expansion were included, whereas those using Maxillary Expansion through Segmented Osteotomy were excluded.

**Result:**

Rapid Maxillary Expansion and Surgically Assisted Rapid Maxillary Expansion cause dentofacial changes, especially in the nasal cavity. Consequently, the nose width enlarges, reducing Nasal Airflow Resistance.

**Conclusion:**

Anteroposterior cephalometric studies show evidence of an enlarged nasal cavity following maxillary expansion.

## INTRODUCTION

The occurrence of maxillary deformities together with respiratory problems - especially nasal block - has been the focus of many researchers who have investigated the possibility that these events are related.^1^, ^2^, ^3^, ^4^

**Figure 1:**
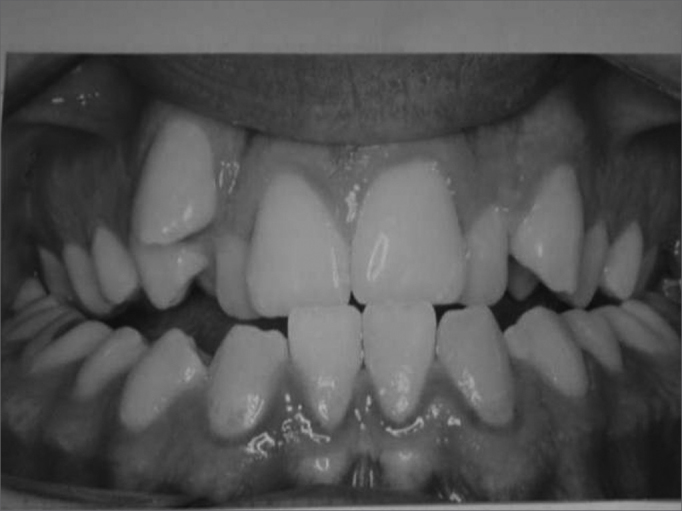
Frontal view of dental occlusion in a patient with maxillary atresia. Note the presence of bilateral posterior crossbite and anterior dental crowding.

The transverse maxillary deficiency is the most frequent maxillary deformity. Patients with this deformity usually presented unilateral or bilateral posterior crossbite and anterior dental crowding (Fig. 1). The distance between the lateral walls of the nasal cavity and the nasal septum is often decreased in the transverse maxillary deficiency. This reduction increases the resistance to nasal airflow and causes nasal respiratory difficulties.^5^, ^6^

Maxillary expansion is a procedure indicated for patients with transverse maxillary deficiency, which is done either orthodontically or surgically.^6^

**Figure 2:**
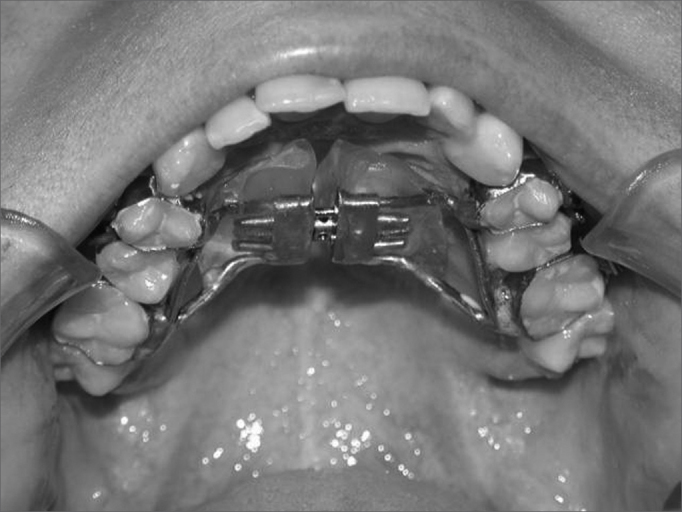
Cemented Hyrax-type expander apparatus (tooth-borne) on teeth before the maxillary expansion procedure.

**Figure 3:**
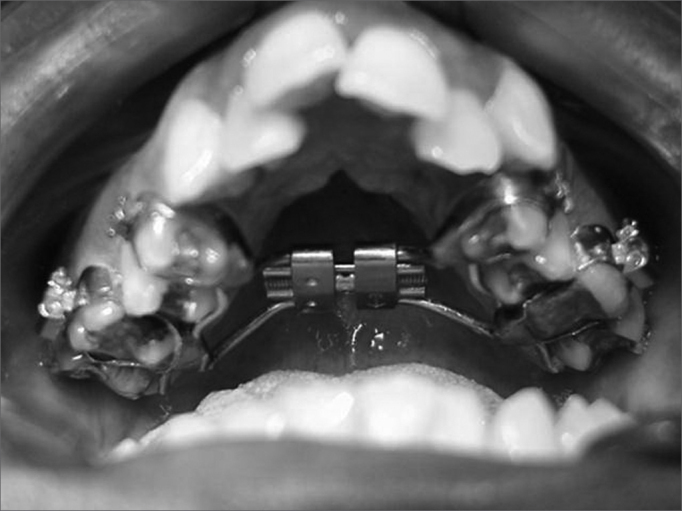
Cemented Haas-type expander (tooth tissue-borne) on teeth before the maxillary expansion procedure.

There are three rapid maxillary expansion methods: orthodontic expansion, orthosurgical expansion and surgical expansion. Indications depend on the patient’s age and the degree of deformity. Orthodontic expansion, also named rapid maxillary expansion (RME), is a procedure whereby an expander is cemented to premolar and upper molar teeth; the desired expansion is obtained by consecutive activation (Figs. 2 and 3). It is an effective procedure for treating maxillary atresia in children and adolescents below age 15 years. After this age - when growth has ceased and bone maturation is complete - the intermaxillary suture is closed, which causes skeletal resistance to the orthodontic procedure only,^7^ requiring associated surgery for any effect. Orthosurgical expansion, also named surgically assisted rapid maxillary expansion (SARME), adds maxillary osteotomies along the skeletal resistance zones to facilitate maxillary expansion by expanders. Many such techniques have been described in the literature.^8^, ^9^, ^10^ The third method for rapid maxillary expansion is to use surgery alone; this procedure is named maxillary expansion by segmented osteotomy. Expansion is attained in this procedure without using any expander; it is done only with segmented osteotomies of the maxilla, and is indicated for transverse deficiencies measuring not more than 7 mm and that are associated with other maxillary deformities requiring surgical correction.^11^

**Figure 4:**
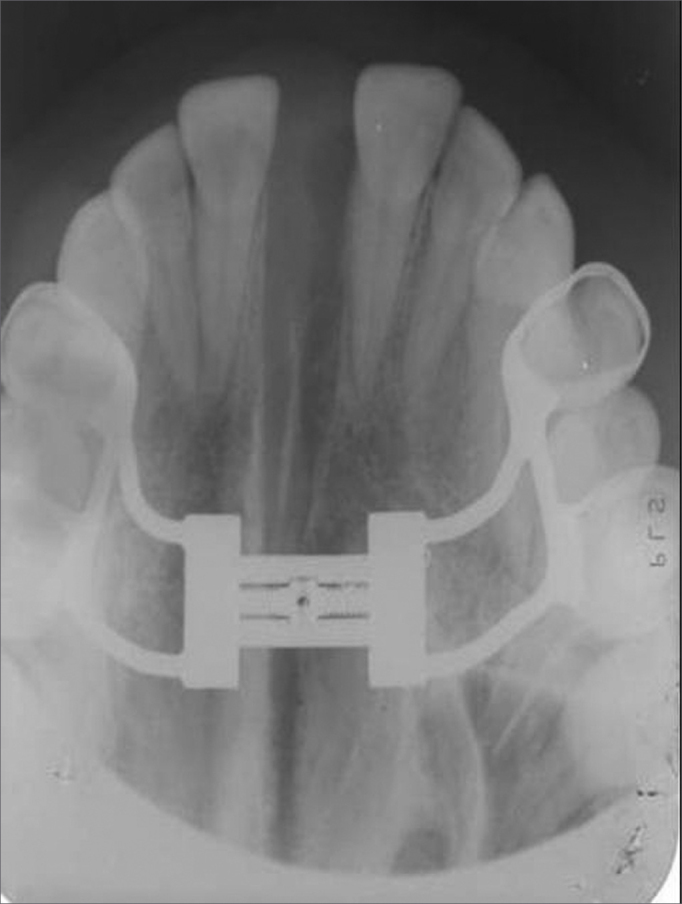
Occlusal radiograph of the maxilla after the maxillary expansion procedure. Note the opening of the midpalatal suture.

When the required amount of maxillary expansion is attained the expander should be blocked and kept in position during a 3 to 6-month retention period, depending on the expansion technique and bone neoformation along the midpalatal suture, which may be monitored radiographically by occlusal maxillary radiographs (Fig. 4). The retention period favor stability of the maxillary expansion procedure, whether orthodontic or surgical.^12^, ^13^, ^14^

During the RME and SARME procedures, expanders cemented on maxillary teeth apply a force that alters the position of the maxilla. The behavior of the maxilla may be observed on occlusal radiographs on the horizontal plane. The maxillae separate along the midpalatal suture, creating a triangular radioluscence with a larger opening in the anterior portion (Fig. 4). The maxillary movement results in an increased transverse diameter in the alveolar arch and the nasal cavity.^4^

The literature contains many papers describing relief of nasal obstruction after opening of the midpalatal suture by maxillary expansion in patients with maxillary atresia.^1^, ^2^, ^15^, ^16^, ^17^, ^18^, ^19^, ^20^ Relief of nasal obstruction occurs in cases where there was narrowing of the antero-posterior portion of the nasal cavity; during the maxillary expansion procedure this region is widened transversally. Such widening increases the airway space in the nasal cavity, resulting in improved nasal patency.^2^

Postero-anterior and lateral cephalometries, tomographies and photographs may be used for assessing the nasal cavity shape following maxillary expansion. Analysis of nasal airway resistance (NAR) and the nasopharyngeal space may be done using rhinomanometry, acoustic rhinometry and nasofibroscopy.

The purpose of this paper was to describe the relation between maxillary expansion, widening of the nasal cavity and NAR, based on a review of the literature.

## REVIEW OF THE LITERATURE

The first documented example of orthodontic correction of maxillary width discrepancies is Angle’s report in 1860. Angle undertook RME using an appliance with an expander screw in youths aged 14 years and found that by turning the expander screw daily, he was able to open the midpalatal suture within two weeks.^21^

Eysel in 1886 - cited by Haas in 1961 - was the first rhinologist to study the effects of RME on the nasal cavity. He found that many changes occurred in the maxillary and adjacent bones during the post-expansion period, and that RME caused a decrease in the NAR. While monitoring the expansion, an increase in the width of the nasal cavity close to the midpalatal suture was found.^1^

The various items that we studied were divided into topics to facilitate the study of nasal cavity alterations resulting from maxillary expansion:

### Changes in the shape and size of the nasal cavity

Wright’s study of 30 patients with nasal respiratory insufficiency in 1911 treated by RME showed, by measuring the width of the nasal cavity with pre- and post-expansion cephalograms, a mean 6.5 mm increase in width following the expansion procedure.^22^

Using occlusal radiographs to assess 40 cases treated with maxillary expansion, Thorne in 1960 found that the increase in nasal width ranged from 0.4 to 5.7 mm (mean - 17 mm) at the end of the procedure. This author also noted that a two-month retention period was required for stability of the increased width.^23^

Anatomically the nasal cavity increases in width immediately after maxillary expansion, particularly of the nasal floor close to the midpalatal suture. Haas made this statement in 1961 based on animal studies, after which he undertook a clinical trial in human beings. Ten patients with nasal insufficiency and maxillary atresia, aged between 9 and 18 years were selected and subjected to RME. Analysis of frontal and lateral cephalometries, photographs and subjective questionnaires answered by patients was done. These authors found that the width of the nasal cavity increased by 2.0 to 4.5 mm, and concluded that improved nasal breathing depended on the severity of the nasal cavity narrowing before maxillary expansion.^1^

Hershey et al.’s study in 1976 aimed to answer issues about changes in the NAR following RME and altered nasal cavity width. Postero-anterior radiographs were done in the pre- and post-expansion period in 17 patients aged between 11 and 14 years undergoing RME. The nasal cavity contour was traced and measured in a postero-anterior radiograph and the maximum diameter of concavities was also measured. The authors found a mean gain of 2.03 mm between pre- and postoperative values of these measures.^24^

Cross et al. in a study published in 2000 compared the transverse measurements of skeletal, dental and nasal structures in patients with maxillary atresia before and after RME, based on digital postero-anterior cephalometric radiographs. Subjects were a group of 25 patients with a mean age of 13 years. The authors found that the mean increase of the nasal cavity width was 1.06 mm.^25^

Bascifti et al. in 2002, aiming to analyze the nasal changes due to maxillary expansion, selected two groups of patients with permanent teeth and maxillary atresia. One group consisted of 15 patients (mean age - 12.1 years) who underwent RME and the other group comprised 15 patients (mean age - 18.4 years) who underwent SARME. Postero-anterior and lateral facial radiographs were used for measuring the width of the nasal cavity and the nasopharyngeal area. These authors found that there was a mean nasal width increase of 3.47 in the RME-treated and 2.93 mm in the SARME-treated group; they concluded that both procedures increased the intranasal volume, and that there was no statistically significant difference between both groups.^17^

Tecco et al. in 2005 studied 55 girls (mean age -8.1 years) who required RME to assess the effects of this procedure on the nasopharyngeal space in children with nasal obstruction. These patients were subdivided into two groups: group 1 consisted of 23 RME-treated patients and group 2 was composed of 22 patients that did not undergo RME, as the control group. Lateral radiographs of the face were done in all patients before surgery and six months postoperatively. The expansion process was able to significantly increase the nasopharyngeal space compared to the control group.^26^

Barreto et al. in 2005 studied the nasal cavity width using postero-anterior cephalometries in 20 patients aged between 7 and 11 years who underwent RME. The mean gain in the transverse measurement of nasal fossae was 2.81 mm.^20^

Machado Jr et al. in 2006 undertook a study to assess cephalometric changes resulting from orthodontic expansion of the maxilla in adults. The sample consisted of 12 patients aged between 18 and 37 years that underwent maxillary expansion. Postero-anterior cephalometric radiographs were made before and immediately after expansion. These authors assessed various points including nasal width, which increased by 1.92 mm and nasal height, which increased by 2.5 mm.^19^

### Changes in the NAR and the breathing pattern

Wertz in 1968 studied two groups of patients with posterior crossbite who were treated with RME for correcting maxillary atresia. Group 1 consisted of four patients (mean age - 11 years) with difficult nasal breathing. Group 2 comprised nine patients (mean age - 12 years) with normal nasal breathing. Nasal airflow was measure at rest, after moderate exercising and during maximum ventilation. A modified facial mask was used to measure the volume of air passing through the nose during air intake and exhalation to compare the volume of nasal air before and after expansion. There was no statistically significant change in nasal airflow within group 2. All group 1 patients had increased nasal air volume during maximum ventilation. The author concluded that opening of the midpalatal suture by RME mainly to increase nasal patency could not be justified, except when there was obstruction located in the antero-inferior portion of the nasal cavity accompanied by transverse maxillary deficiency.^2^

Hershey et al. in 1976 assessed RME-related changes in the NAR and their long-term stability. A clinical study was done of 17 patients aged between 11 and 14 years, described by their parents as mouth-breathing children. All underwent RME, having used the expander for three months. The NAR was measured prior to the treatment, after maximum expansion and after three months retention. The NAR was calculated according to the air pressure during breathing, measured by a device with two 1.5 mm diameter catheters, one in the oropharynx and the other adapted to a nasal mask. The authors concluded that RME, if well indicated, is not only effective for increasing the width of the maxillary arch, but also for reducing the NAR to normal nasal breathing levels.^24^

Warren et al. conducted a similar study in 1987 to compare the NAR before and after maxillary expansion in two groups of patients; these authors used the NAR measuring method created by Hershey et al., in which one group consisted of RME-treated patients and the other group comprised patients who had undergone surgical maxillary expansion. The results showed that both procedures (RME and surgical maxillary expansion) improved the nasal airway. However, about 1/3 of patients in both groups did not improve sufficiently abandon mouth breathing.^27^

Hartgerink et al. measured the NAR in a 1987 study by comparing a group of 38 patients (mean age - 11.75 years) who underwent RME with a control group consisting of 24 subjects (mean age - 12 years) who did not undergo RME. Nasal airway resistance was measured under four conditions: in a natural state; with nostrils dilated by a Tygon tube; while administering a decongestionant; and with nostril dilatation associated with a decongestionant. The four conditions were investigated before treatment, after treatment, and one year later. The authors concluded that there was a significant mean reduction in the NAR after RME as measured in the natural state, and that this change was stable one year following maximum expansion. This study showed no significant decrease in the NAR after expansion when using a decongestionant. This study also noted that RME could not offer a conclusive prognosis about NAR reduction due to wide individual variations.^5^

Doruk et al. in 2004 used acoustic rhinometry to analyze the NAR in patients undergoing RME. The sample consisted of 22 RME-treated children (mean age - 9 years) with maxillary atresia. The NAR was measured before and after expansion and at the end of the post-expansion retention period. Acoustic rhinometry was done in each patient using and not using nasal decongestionants. The results showed a mean decrease in the NAR of 0.024 cm H20/L/m. The authors underlined that these findings were not sufficient for indicating RME with the aim to improve the NAR, since their sample was small, there was no control group and follow-up was short.^28^

Bicakai et al. in 2004 used acoustic rhinometry to assess the effects of RME on the least cross-sectional transverse nasal area by measuring the NAR in patients. These authors studied 29 patients divided into two groups according to their skeletal maturation. Group 1 consisted of 16 patients treated early (mean age - 11 years), and group 2 was composed of 13 patients (mean age - 13 years). Acoustic rhinometry recordings were done before treatment, after expansion and after a 3-month retention period. There was an increase in the least cross-sectional nasal area, as follows: on average 0.34 mm in group 1 patients, and on average 0.19 mm in group 2 patients. This difference was not statistically significant, and the authors concluded that RME increases the least cross-sectional nasal area in all patients.^18^

Babacan et al. in 2006 undertook a study composed of two groups of patients with maxillary atresia and bilateral posterior crossbite. The first group consisted of 10 patients (mea age - 12.3 years) that underwent RME; the second group was composed of 10 patients (mea age - 18.7 years) that underwent SARME. The authors used acoustic rhinometry to assess the effects of maxillary expansion on the nasal volume; measurements were done with and without nasal decongestionants before the treatment and after the retention period. The nasal volume increased significantly in both groups, but was not affected by decongestionants. ^29^

### Subjective improvement in nasal breathing

Timms in 1984 conducted a retrospective study of 240 patients aged from 6 to 29 years, making a subjective analysis using questionnaires and case monitoring, and concluded that RME is a simple procedure for improving cases of narrow nasal cavities, resulting in subjective improvement of nasal breathing.^15^

The same author undertook another study in 1987 to analyze 300 patients using questionnaires. The mean age of these patients was 13 years, and all had posterior crossbites. Of 300 patients, 178 had breathing difficulties (71 had repeated upper airway infection, 63 had allergic rhinitis and 34 had asthma). The authors reported that after RME the improvement rate was 82% in cases that had upper airway infection, 60% in cases that had allergic rhinitis, and 47% in cases that had asthma.^30^

Doruk et al.’s previously mentioned study in 2004 includes an assessment of patient’s opinions about the questionnaire analysis. These authors reported that 59% of 22 patients undergoing RME had a subjective improvement of nasal breathing after maxillary expansion.^28^

Ribeiro et al. in 2006 assessed 10 patients (mean age - 17 years) that underwent SARME. In interviews of these patients revealed that 60% reported improved nasal breathing.^31^

## DISCUSSION

The first description in the medical and dental literature about the treatment of transverse maxillary deficiencies by maxillary expansion procedures was made in 1860 by Angle.^21^ RME and SARME are well-established procedures in orthodontics and buccomaxillofacial surgery and are widely used in the treatment of transverse maxillary deformities. The effects of RME and SARME on the nasal cavity and the breathing pattern started to be investigated around 1886, and remain objects of study and diverging opinions to this day.^1^, ^2^, ^3^, ^4^

For many years, various published papers described maxillary expansion procedures and their favorable effect on nasal breathing, albeit based on subjective data. Only in 1961 did Haas describe the effect of RME in opening midpalatal suture and displacing the walls of the nasal cavity laterally and away from the nasal septum. The floor of the nasal cavity is displaced as alveolar processes tilt laterally and the free margins of the horizontal palatal process are displaced inferiorly. The results would be increased intranasal area.^1^

**Chart 1:**
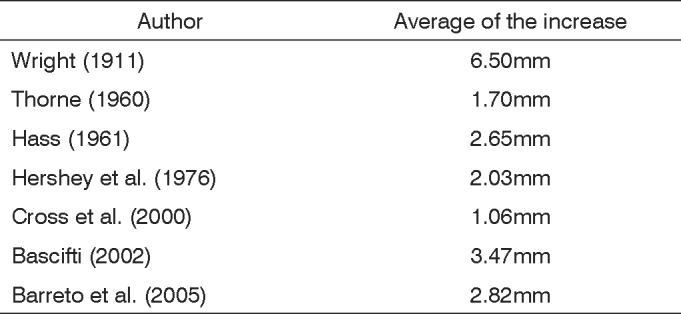
Mean increase of nasal cavity width measured in postero-anterior cephalometries after rapid maxillary expansiona

Postero-anterior cephalometry is one of the best methods for demonstrating nasal cavity increases after maxillary expansion. Various papers have objectively demonstrated increases in the nasal cavity using pre- and post-treatment (maxillary expansion) cephalometry.^2^, ^4^, ^5^, ^23^, ^24^, ^27^, ^28^, ^29^ Anatomically, there is widening of the nasal cavity following maxillary expansion, particularly of the nasal floor close to the midpalatal suture. This increase varies among authors (Chart 1), depending on the patient’s age and the procedure. When studies demonstrated that maxillary expansion procedures increased the width of the nasal cavity, research focused on their effects on the nasal breathing pattern. If maxillary expansion is expected to increase the width of the nasal cavity, it should also improve airflow by reducing the NAR. Researchers used various methods to study the pre- and post-maxillary expansion period to analyze nasal breathing. Questionnaires answered by patients provide a subjective assessment of improvements in nasal breathing. Such subjective analyses have shown that always more than 50% of patients undergoing maxillary expansion report improved nasal breathing.^15^, ^28^, ^30^, ^31^

Although rhinomanometry and acoustic rhinometry do not provide reliable absolute numbers to faithfully express the physiology of nasal breathing and respiratory function, these tests have been widely used for assessing the effects of RME on the NAR.^16^ Nasal airflow and pressure have been investigated before and after RME, suggesting that maxillary expansion increases the nasal volume and reduces the NAR.^2^, ^4^, ^5^, ^6^, ^18^, ^24^, ^27^, ^28^, ^29^

There remains some controversy in the literature about the relation between maxillary expansion and the nasal respiratory pattern. Warren et al. have stated that the increased nasal flow resulting from a wider intranasal space produced by RME is not enough to change mouth breathing into nose breathing; an increased NAR may be associated - according to many authors - to turbinate hypertrophy, nasal polyps, adenoid hypertrophy and a deviated nasal septum, over which RME would have little effect. In their studies, these authors have stated that RME only for increasing the nasal breathing capacity may not be justified.^27^ There are, however, other authors that have defended RME as an attempt to improve nasal breathing even in those patients with no evident crossbite, as long as these patients have maxillary atresia, hypertrophied turbinates and mouth breathing.^16^ Although many published papers have shown that nasal breathing improves after RME, Graber believes that such improvement is temporary. Graber suggests that an important factor is that at age 12 years, a child has much more rhinopharyngeal and oropharyngeal lymphoid tissue compared to adults. These tissues block nasal breathing in childhood; with growth, however, lymphoid tissues regress spontaneously, which automatically improves nasal breathing.^32^

## CONCLUSION

Pre- and post maxillary expansion antero-posterior cephalometric radiographs provide concrete data that make it possible to visualize the skeletal changes in the nasal cavity inherent to maxillary expansion. These studies show that the width of the nasal cavity is increased following maxillary expansion.

The effects of RME on the nasal airway and the nasal respiratory pattern are very important. These effects depend on the existence or not of nasal obstruction and on its cause, location and severity. In general, both the subjective assessments by patients and the objective evaluations that use methods for measuring the nasal airflow and the NAR demonstrate that there is significant improvement in nasal breathing following maxillary expansion. There is, however, wide variation in individual responses to RME; this procedure, therefore, does not necessarily predict a reduction in the NAR.
